# Allogeneic CAR-T Cells: More than Ease of Access?

**DOI:** 10.3390/cells7100155

**Published:** 2018-10-01

**Authors:** Charlotte Graham, Agnieszka Jozwik, Andrea Pepper, Reuben Benjamin

**Affiliations:** 1 Department of Haematological Medicine, King’s College London, London SE5 9NU, UK; ajozwik@kcl.ac.uk; 2 Department of Haematology, King’s College Hospital NHS Foundation Trust, London SE5 9RS, UK; 3 Department of Clinical and Experimental Medicine, Brighton and Sussex Medical School, University of Sussex, Falmer BN1 9PX, UK; A.Pepper@bsms.ac.uk

**Keywords:** CAR-T cells, cancer immunotherapy, gene editing

## Abstract

Patient derived anti-CD19 chimeric antigen receptor-T (CAR-T) cells are a powerful tool in achieving a complete remission in a range of B-cell malignancies, most notably B-acute lymphoblastic leukaemia (B-ALL) and diffuse large B-cell lymphoma (DLBCL). However, there are limitations, including inability to manufacture CAR-T cells from the patient’s own T cells, disease progression and death prior to return of engineered cells. T cell dysfunction is known to occur in cancer patients, and several groups have recently described differences in CAR-T cells generated from chronic lymphocytic leukaemia (CLL) patients compared with those from a healthy donor. This is thought to contribute to the low response rate in this disease group. Healthy donor, gene-edited CAR-T cells which do not require human leucocyte antigen (HLA) matching have the potential to provide an ‘off the shelf’ product, overcoming the manufacturing difficulties of producing CAR-T cells for each individual patient. They may also provide a more functional, potent product for malignancies such as CLL, where T cell dysfunction is common and frequently cannot be fully reversed during the manufacturing process. Here we review the potential benefits and obstacles for healthy donor, allogeneic CAR-T cells.

## 1. Introduction

Immune surveillance is the process by which the immune system is believed to detect and destroy cancerous cells before clinical malignancy develops. Therefore, for a patient to develop a tumour their malignant cells must have evaded immune elimination. Immunotherapy-based treatment strategies have been around for many years and are designed to enhance and manipulate immune responses to cause tumour regression. Chimeric antigen receptor-T (CAR-T) cells are one such emerging immunotherapy whereby T cells are genetically modified to recognise a particular antigen that the tumour cells are known to display. These engineered T cells express an artificial receptor consisting of an antigen recognition domain, typically a single chain variable fragment (scFv), a co-stimulatory domain (usually 4–1BB or CD28) and an intracellular CD3ζ signaling domain. The majority of CAR-T cell studies have used autologous T cells collected from patients which are transduced, usually with a viral vector (lentivirus or retrovirus), introducing the CAR construct. Transduced T cells are expanded ex vivo and then infused back into patients following lymphodepleting chemotherapy. This technology has overcome several strategies that malignant cells adopt to hide from the immune system and avoid immune destruction. Tumour cells can downregulate human leucocyte antigen (HLA) expression, but unlike a normal T cell receptor the CAR does not require the HLA complex for antigen recognition and binding. In addition, tumour cells have reduced expression of co-stimulatory molecules and increased expression of inhibitory molecules impairing T-cell response [1]. The co-stimulatory domain within the CAR construct bypasses the need for tumour cells to express co-stimulatory molecules and to some extent ameliorates the effect of immune checkpoint expression.

**Figure 1:**
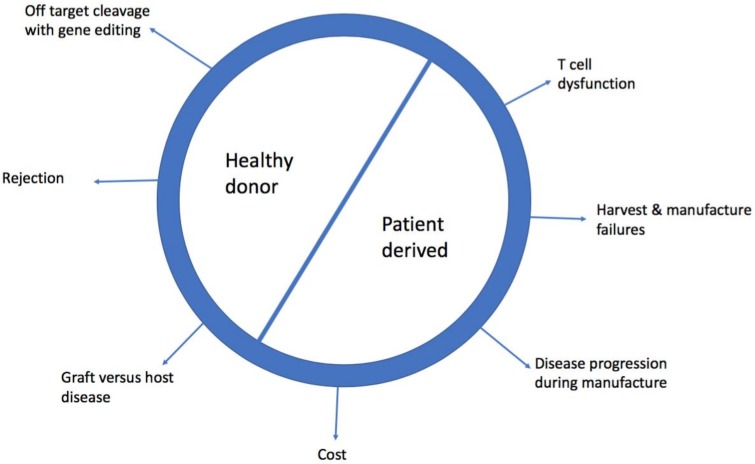
Challenges of using healthy donor and patient derived chimeric antigen receptor-T (CAR-T cells).

The clinical efficacy of this technology has been proven, with stunning complete remission rates (CR) in B-acute lymphoblastic leukaemia (>80% of patients) [2,3] and promising early data in diffuse large B-cell lymphoma (DLBCL) (>40% CR) [4,5]. However, there are major limitations to accessing this technology. Currently, it is a bespoke product made for individual patients, therefore the time to manufacture can prevent access, as can the cost. Furthermore, the T cells used as starting material from patients are likely to have developed cancer associated T cell dysfunction which may not be reversible [6]. Healthy donor allogeneic CAR-T cells can be derived from patients’ previous haematopoietic stem cell transplant (HSCT) donor, who they are HLA matched to or from gene edited cells which have been modified to allow them to be given to non-HLA matched patients. Here we review the limitations of patient derived (PD) CAR-T cells and discuss the potential benefit and challenges of healthy donor (HD) CAR-T cells. Figure 1 summarises the obstacles of using PD and HD CAR-T cells.

## 2. Limitations of Patient Derived, Autologous Chimeric Antigen Receptor-T (CAR-T Cells)

### 2.1. Cost

The first FDA approved PD CAR-T cell product, Tisagenlecleucel, from Novartis, licenced to treat paediatric and young adult B-ALL, is priced at $475,000 US dollars per patient [7]. KITE/Gilead anti-CD19 CAR-T cell product, Axicabtagene ciloleucel, for DLBCL, is being marketed at $373,000 USD per patient [8] and Novartis have matched this price when using their product in the setting of lymphoma. This cost is purely for the T cell manufacture and excludes other aspects of patient care, which are likely to be high as patients frequently require admission to intensive care.

### 2.2. Harvest and Manufacturing Failures

It is not always possible to harvest and manufacture adequate lymphocyte numbers from patients who are typically lymphopenic from their disease or previous chemotherapy. It is likely that severely lymphopenic patients are those most heavily pre-treated and therefore may represent a particularly poor risk group. The multicentre ZUMA-1 study, using Axicabtagene ciloleucel, in DLBCL, only included patients with a lymphocyte count > 100/μL [5]. Published response rates often exclude patients with an insufficient lymphocyte harvest. Indeed, the multinational ELIANA study, in which Tisagenlecleucel was used to treat paediatric and young adult B-ALL, lists having an adequate apheresis product received by the manufacturer as one of the criteria for enrolment [2]. Despite this stipulation manufacturing failures still occur. In the ELIANA study, 7.6% of patients did not receive the cells due to ‘product related issues’ [2], this was even higher using the same CAR construct in adults with DLBLC or follicular lymphoma (FL) with 13.2% manufacturing failure cases (all patients with manufacturing failure had an absolute lymphocyte count < 300/μL) [4].

### 2.3. Product Variability and Quality Control

As cells are manufactured for each individual patient from their apheresis collection it is not possible to produce a completely standardised end product. Attributes which are thought to be key such as cell number, viability, CAR expression and memory phenotype can be monitored but will not be entirely consistent from product to product [9]. Manufacturing processes are being optimised in an attempt to ensure the quality of the product, for example by using high titre purified lentivirus [9] but problems of viability and varying phenotype remain challenging.

### 2.4. Disease Progression During Manufacture

Disease progression during manufacture is a significant barrier to accessing this technology. In the JULIET study, where Tisagenlecleucel was used to treat relapsed, refractory DLBCL and FL, 13% of patients never received the CAR-T cell product due to disease progression [4]. Using the same product in paediatric and young adult B-ALL, 7.6% of enrolled/leukapheresed patients died before receiving the CAR-T cells [2]. Manufacture typically takes 14–21 days, but may decrease as improvements are made in production.

### 2.5. Contamination with Tumour Cells

Patients with refractory disease often have circulating leukaemia cells in the blood which are harvested during leukapheresis. These tumour cells are killed by transduced T cells during the manufacturing process. However, cases have been reported where the leukaemia cell is transduced by the CAR construct, which when expressed is thought to block its own CD19 antigen, preventing engagement by CAR-T cells. This has lead to relapsed leukaemia cells which express the CAR construct [10].

### 2.6. T Cell Dysfunction

Chronic lymphocytic leukaemia (CLL), like B-ALL and DLBCL is a CD19 expressing B cell malignancy with a similar anatomical distribution of disease (bone marrow and lymphoid organs). However, results from clinical trials using anti-CD19 CAR-T cells have been disappointing with only 26% of patients having a sustained response [11]. Investigators have been unable to identify tumour related characteristics which predict response to CAR-T cell therapy [12]. T cell dysfunction is well described in CLL and is more marked in advanced disease [13]. Fraietta and colleagues carried out a thorough evaluation of the apheresed T cells and CAR-T cell product from responding (CR) and non-responding (NR) CLL patients [12]. They found expansion during manufacture was a simple predictor of response and correlated with proliferation in vivo which is necessary for CAR-T cells to have sustained anti-tumour activity. When the CAR-T cell product from CR patients was compared to those from NR patients in an immunodeficient murine xenograft model (NSG mice engrafted with the CD19 positive NALM-cell line), CR patient derived CAR-T cells expanded more and caused superior tumour regression. This lead to prolonged survival compared to those treated with NR patient derived CAR-T cells, suggesting that it is the T cell product as opposed to tumour related factors which were influencing response [12].

A comparison of the gene expression profile of NR and CR patient derived CAR-T cells found those from NR patients expressed more exhaustion markers and genes associated with effector differentiated T cells [12]. In contrast, those derived from CR patients had a signature consistent with an early memory stage phenotype [12]. In this model, HD CAR-T cells were used as a control and performed at least as well as the CAR-T cell product from CR patients. Mice treated with HD CAR-T cells had the longest survival and showed sustained tumour regression [12] supporting the hypothesis that HD CAR-T cells may provide a more functional anti-leukaemia product, at least in the context of CLL and other malignancies characterised by T cell dysfunction. Interestingly, the authors also found a higher frequency of CD27+CD45RO−CD8+ T cells before manufacture correlated with response [12]. Screening and selecting healthy donors with high frequency of these cells is therefore an appealing therapeutic strategy that may improve outcome.

A study by Hoffman et al. compared the phenotype and expansion of CAR-T cells from untreated CLL patients with those from HDs [14]. CAR-T cells from untreated CLL patients might be expected to show less T cell dysfunction than those from the heavily pre-treated, relapsed/refractory patients receiving CAR-T cells in clinical trials. The numbers in each group were low (*n* = 3) but there was greater expansion in naive CAR-T cells (CD45RA+CCR7+) from HDs as compared to untreated CLL patients, who had greater expansion in effector CAR-T cells (CD45RA+CCR7−) [14]. Conflicting results were found by another team but their expansion time following transduction was much shorter at 5–8 days [15]. PD1 expression was significantly higher on naïve and central memory CAR-T cells from untreated CLL patients as compared to HDs [14]. Fraietta et al. describe a CD8+CAR+CD27+PD1− population of cells as an important predictor of response in patients. In functional assays, removal of this population resulted in lack of tumour control [12].

T cell dysfunction is a clear risk factor for B-cell lymphoma [16]. Post-transplant lymphoproliferative disorder occurs in patients who have received immunosuppressive drugs to impair their T cells (to prevent graft rejection). In addition, HIV infection also predisposes to DLBCL, Burkitt’s lymphoma and other malignancies [17]. Furthermore, it has previously been described that DLBCL patient derived T cells proliferate less in response to polyclonal activation compared to those from a healthy donor [18]. T cell defects have also been reported in myeloma patients and some solid organ malignancies [19]. Whether this translates into less functional CAR-T cells in malignancies besides CLL needs to be evaluated.

HD allogeneic CAR-T cells could potentially provide an alternative source of CAR-T cells for patients in whom harvest failures occur and in those with rapidly progressive disease who cannot wait for autologous CARs to be manufactured. Additionally, HD CAR-T cells may overcome the problems of T cell dysfunction seen in malignancy and provide a more potent product. One donor could provide therapeutic cells for multiple patients, with a standardised treatment being manufactured at reduced cost. Donors with a T cell phenotype associated with superior CAR-T cell function could be identified maximizing the quality of the product. Redosing for the same patient can also be done more readily with HD CAR-T cells. However, there are challenges to using HD CAR-T cells that still need to be overcome.

## 3. Barriers to Allogeneic, Healthy Donor CAR-T cells

### 3.1. Graft Versus Host Disease (GVHD)

Graft Versus Host Disease (GVHD) results from donor derived T-cells recognising HLA mismatch via the T cell αβ receptor (TCRαβ) and attacking patient’s tissues. It can be fatal even in the HLA matched donor setting, as minor mismatches may still provoke the response, and is a major complication of HSCT. It can also occur following transfusion, but due to widespread leucodepletion of blood products and irradiation for at risk groups (generally those who are heavily immunosuppressed e.g., after purine analogue chemotherapy), it is now rarely seen. To make HD CAR-T cells safe, GVHD must be prevented.

### 3.2. Rejection of CAR-T Cells

A patient’s T-cells will recognise infused non-HLA matched T-cells as foreign and reject them. HLA matching has reduced graft rejection in HSCT. Not all patients have a fully matched donor and it would be desirable to have a readily available cellular product suitable for all patients independent of HLA type. Intensification of patient lymphodepletion may be sufficient to allow HD CAR-T cells to expand and clear malignant cells, prior to host immune recovery. To enable this, several strategies are being deployed to make donor T-cells resistant to lymphodepleting agents.

## 4. Strategies to Deploy Allogeneic CAR-T Cells

### 4.1. Manufacture of CAR-T Cells from Previous Allogeneic Haematopoietic Stem Cell Transplant (HSCT) Donor

HSCT is the standard of care for high risk B-ALL patients with an HLA matched donor. It is not uncommon for adult patients to relapse post HSCT, therefore clinical trials have evaluated the use of CAR-T cells derived from the same donor. As these donors are HLA matched to the patient, CAR-T cells generated from them are less likely to cause GVHD and, as they are identical to the previously transplanted haematopoietic compartment, they should not attack the graft. Early results show minimal GVHD in contrast to standard Donor Lymphocyte Infusion (DLI) [20] where the rate of acute GVHD is 40–60% [21]. One study reported no acute GVHD at all, although no lymphodepletion was given, so Tregs would have persisted, ameliorating GVHD and potentially the CAR-T cell activity. This strategy resulted in fairly low anti-leukaemia activity (40% remission rate) [20].

The type of co-stimulatory domain used in the CAR-T cell construct appears to impact on the degree of GVHD, with a reduction when a CD28 co-stimulatory domain is used. It is hypothesised that donor derived CAR-T cells with a CD28 co-stimulatory domain receive signals both via the CAR and the TCRαβ resulting in increased exhaustion and cell death as compared to CAR-T cells receiving stimulation via a 41-BB co-stimulatory domain and the endogenous TCRαβ [22,23].

While HSCT donor derived CAR-T cells provide an alternative source of cells for a selected patient group, and may overcome T cell dysfunction, they are not ‘off the shelf’. They still need to be manufactured for each individual patient, whose disease must be controlled during this process. 26% of patients consenting to the NCI/NIH study for CD19 positive B cell malignancies never received the CAR-T cell infusion due to progressive disease and/or medical complications [20]. What is more, allogeneic HSCT is rarely used as a treatment strategy in relapsed DLBCL or CLL and certainly would not be a practical ‘bridge’ to providing an allogeneic CAR-T cell product.

### 4.2. ‘Off the Shelf’ Virus Specific CAR-T Cells

Unlike HSCT donors, third party viral specific (VS) T cell donors are only partial HLA matched (1–4 alleles) to patients. These cells have been used to treat viral infections following transplantation [24]. Despite HLA mistmatches they only show minimal GVHD and in responding patients persisted between 14 and 90 days [25]. They are therefore an appealing source of cells for manufacturing allogeneic CAR-T cells [26]. However, response rates using HSCT donor derived VS CAR-T cells have been disappointing with 1 out of 6 patients treated achieving a CR [27] and the efficacy of using only partially HLA matched VS CAR-T cells has not yet been demonstrated. The requirement for partial HLA matching would also not make these a truly universal product.

### 4.3. Gene-Edited Healthy Donor CAR-T cells

A universal ‘off the shelf’ CAR T cell product could eliminate a lot of the problems of harvest and manufacture failures associated with their autologous and HSCT derived counterparts. Although they are not without their challenges, recent technological advances have been promising in overcoming many of these.

To prevent GVHD the TCRαβ receptor can be knocked out (KO) by different gene editing techniques. TCRαβ is a heterodimer and both alpha and beta chains need to be present for it to be expressed. A single gene codes for the alpha chain (TRAC), whereas there are 2 genes coding for the beta chain, therefore TRAC loci KO using nucleases is the choice strategy for removing TCRαβ expression.

Nucleases act as ‘molecular scissors’ for editing the genome. They use the binding of proteins or guide RNA (gRNA) to a specific target DNA sequence and then cut it using a nuclease domain. When they cut DNA it forms a double stranded break (DSB) and due to error prone non-homologous end joining (NHEJ) the gene is disrupted. Table 1 summarises the gene editing techniques used to KO TCRαβ for HD CAR-T cell production. Gene editing techniques do not reach 100% knockout and even with depletion of TCRαβ+ cells, small numbers remain which could theoretically cause GVHD.

Most strategies to produce gene edited HD CAR-T cells use two steps whereby the CAR is introduced with a viral vector and the TRAC loci disrupted using a nuclease e.g., zinc finger nucleases (ZFNs) or transcription activator like effector nucleases (TALENs). Using this method, similar to PD CAR-T cells, the genes encoding the CAR construct integrate in a semi random fashion with variable expression levels.

Eyquem et al. developed a technique whereby the CAR construct is incorporated into the TRAC loci, which introduces the CAR and prevents endogenous TCRαβ expression in one step [28]. Additionally, this allows expression of the CAR to be controlled by the TRAC locus resulting in more uniform expression and prevention of tonic signalling [28]. The authors used a guide RNA targeting the TRAC loci and Cas9 mRNA, to cut it. To introduce the CAR into the disrupted TRAC site they used an adeno-associated virus with homology arms to the cut loci carrying CAR cDNA [28]. In a murine xenograft model, using the B-ALL cell line NALM-6, the authors found that CAR-T cells produced by this method outperformed CAR-T cells produced by traditional viral transduction. Mice survived longer and had better tumour control. CAR-T cells also showed less terminal differentiation and lower expression of the exhaustion markers PD1, LAG3 and TIM3 [28]. Similarly, another group, this time using a homing endonuclease to disrupt the TRAC loci, introduced the CAR into the cut site with an adeno-associated virus [29].

A different approach linked CAR expression with TRAC disruption by clustered regularly interspaced short palindromic repeats (CRISPR)/Cas9 without directly incorporating the CAR into the TRAC loci. sgRNA sequence for CRISPR was included in the lentiviral vector carrying the CAR. Cas9 mRNA was then electroporated into transduced cells. TRAC disruption therefore only occurred in cells successfully transduced with the CAR (and the sgRNA). After magnetic bead depletion to remove residual unedited cells expressing TCRαβ, the percentage of CAR+ cells was much higher than current methods, at >99% [30]. These cells showed superior anti-tumour efficacy in a murine xenograft model than unedited CAR+ cells and less PD1 expression [30]. Using this technique the authors produced enough CAR+ cells to dose 20 adult patients from a single leukapheresis of a healthy donor [30]. Advances in gene editing techniques may not only improve access to CAR-T cells but produce CAR-T cells with superior anti-tumour efficacy.

### 4.4. Non Gene Edited Mismatch CAR-T Cells

An alternative strategy to prevent GVHD, currently at the developmental stage, is the modification of CAR-T cells to express an inhibitor of TCRαβ signalling. Gilham et al. report the use of a truncated form of CD3ζ as a TCR inhibitory molecule, which in an NSG mouse model did not cause GVHD and retained CAR activity [31]. A clinical trial with this product is planned in colorectal cancer, with the CAR construct targeting natural killer group 2D (NKG2D) ligands [31]. Solid tumours to date have not had the dramatic response rates to PD CAR-T cells which have been seen in haematological malignancies. There are several reasons for this, including heterogenous antigen expression and the immunosuppressive tumour microenvironment [32]. Solid tumours are known to be associated with T cell dysfunction [33] and this trial will help explore if HD CAR-T cells can produce better responses.

### 4.5. To Prevent CAR-T Cell Rejection

Intensification of lymphodepletion may be sufficient to allow HD CAR-T cells to expand and clear malignant cells prior to host immune recovery. Several strategies are being deployed to make donor T-cells resistant to lymphodepleting agents such as Alemtuzumab (monoclonal anti-CD52) [34] and purine analogues [35]. UCART19 has had CD52 KO with TALENs thus producing resistance to Alemtuzumab [34]. This has shown promise with expansion and anti-leukaemia activity of these cells seen in patients with B-ALL [36]. Two clinical trials are currently evaluating UCART19 in adult B-ALL (CALM, NCT02746952) and Paediatric B-ALL (PALL, NCT02808442).

Gene editing has additionally been used to prevent expression of HLA class I molecules on CAR-T cells. Torikai et al. used ZFNs to knock out the HLA-A locus [37] but a more decisive approach was adopted by Ren et al. to target Beta-2 microglobulin (B2M), which is required for HLA class I expression [38]. This approach prevents host TCRαβ cells recognising donor CAR-T cells as foreign via HLA class I. However, activated T cells also express HLA Class II which might stimulate rejection. NK cells also target cells with reduced HLA expression and represent a further challenge to preventing CAR-T cell rejection [38,39]. Using the CRISPR/Cas9 system researchers have simultaneously KO TCRαβ, B2M and the immune-checkpoint PD1 which showed enhanced anti-tumour activity in a NALM6 NSG murine model without inducing GVHD [38]. In China, a clinical trial is recruiting where HD CAR-T cells have both the TRAC and B2M gene disrupted by CRISPR/Cas9 (NCT03166878) although outcome data is not yet available. Data on long term persistence and function of CAR-T cells which have had either TRAC or HLA expression disrupted is not yet available and may be key to their success.

### 4.6. Challenges of Gene Editing

Gene editing is revolutionising the possibilities for cellular technologies, but such highly manipulated products come with unknown risks. Off target cleavage of genes can occur, where multiple sites are cut, new translocations can occur. In UCART19 recombination between CD52 and TRAC loci is seen, but does not appear to cause a proliferative advantage (Poirot, et al. 2015). Researchers must remain vigilant to introducing oncogenic potential into edited cells. This issue was highlighted by Fraietta et al. who reported the incidental disruption of the *TET2* gene due to lentivirus coding for the CAR construct integrating at this site. This resulted in a proliferation and survival advantage of CAR-T cells with this disruption as compared to those without [40]. Inserting the CAR construct into the TRAC loci may actually reduce the risk of insertional mutagenesis [29] however, with new gene editing technologies going into patients, long term safety data is lacking.

### 4.7. Inducible Pluripotent Stem (iPS) Derived CAR-T Cells

To remove the requirement for repeated donations by healthy volunteers and create a bank of different HLA type CAR-T cell lines, researchers have generated induced pluripotent stem (iPS) CAR-T cells [44]. Transducing HD T cells with reprogramming factors can restore pluripotency. The CAR construct is then introduced by a lentiviral vector. iPS CAR-T cells are capable of self renewal and additional gene editing steps such as TRAC KO are being proposed, which could provide a continuous supply of universal CAR-T cells [44]. The safety and efficacy of this approach in humans is not known. Once re-differentiated T cells had a phenotype most resembling gamma delta (γδ) cells, they had lower levels of CAR expression and in an immunodeficient xenograft mouse model, resulted in shorter survival than mice treated with CAR-T cells from normal αβ T cells [44]. This is an intriguing future source of cells, but numerous obstacles need to be overcome.

## 5. Discussion

Autologous PD CAR-T cells have shown huge potential in treating patients with CD19 positive malignancies. However, relapsed refractory cancer patients in whom this therapy has proven so hopeful, rarely have stable disease. They must wait for an apheresis slot to collect their own lymphocytes and the manufacturing time for the CAR-T cell product to be made. Patients with resistant disease are likely to have received multiple lines of intensive chemotherapy, impairing their own lymphocyte count and function. Furthermore, T cell dysfunction can occur in cancer patients even in the absence of therapy as exemplified in CLL. CAR-T cells from previous HSCT donors may provide an alternative supply of cells for a small subset of patients but still need to be produced as a bespoke product. Healthy donor, gene edited, allogeneic CAR-T cells could provide a readily available, ‘off the shelf’ product for patients. It is hoped this will decrease the cost per patient, however, gene editing will add to the complexity of the manufacturing process. HDs may provide a more functional T cell product and could be screened for a T cell phenotype linked with more potent anti-leukaemia activity such as CD8+CD27+CD45RO– associated with improved response in the setting of CLL [12]. Donors could then provide CAR-T cells for multiple patients and a standardised high quality product manufactured. Gene editing to knockout the TCRαβ receptor is an attractive approach to prevent GVHD that is being deployed by several groups using ZFNs [41], TALENs [34,36,42], CRISPR/Cas9 [28,30,38,43], megaTAL nucleases [43] and engineered I-CreI homing endonucleases [29]. Although these techniques do not edit 100% of cells, antibody coated magnetic bead depletion has been used to purify the end product to >99% TCRαβ− cells, thus enabling their use in patients [36]. Pre-clinical work in murine models suggests that gene editing techniques that knock out the TRAC loci, incorporating the CAR construct into that site, may actually produce a more functional CAR-T cell product than current methods used to generate PD CAR-T cells.

Rejection of CAR-T cells can occur in the PD CAR-T cell setting and is likely to be a greater challenge with HD CAR-T cells. Murine derived elements of the CAR construct can be particularly immunogenic. Lymphodepleting chemotherapy is required for successful expansion of infused cells even when they are autologous. This is thought to be required to reduce the immunosuppressive host cells such as regulatory T cells (Tregs). Intensification of lymphodepletion may allow non-HLA matched cells to be given. In this context, proof of principle has been shown with the use of Alemtuzumab which allows expansion of UCART19 (which has CD52 KO) [36]. When the host immune system recovers, it would be expected to reject HLA mismatched cells. It is not yet clear how long CAR-T cells need to persist to ensure a deep remission is achieved and this is likely to vary between the different types of malignancy treated. Knocking out B2M, which is required for HLA class I expression, may allow CAR-T cells to ‘hide’ from the host TCRαβ cells. This may be hampered by activated T cells expressing HLA class II and NK cells targeting cells with reduced HLA expression. Therefore, at present it seems unlikely that intensification of pre-conditioning chemotherapy can be avoided. Whether the expected increased risk of infection for patients is manageable needs to be carefully assessed in clinical trials. Certainly, there is optimism that gene editing can provide quick access to CAR-T cell therapy at reduced cost, for a much wider group of patients who have the most aggressive and resistant disease. It may also provide a more functional, potent, anti-cancer therapy.

## Figures and Tables

**Table 1:** Summary table of different gene editing techniques used to disrupt T cell αβ receptor (TCRαβ) expression.

	Binding Region	Nuclease	Cutting Efficiency	Reference
Zinc finger nucleases (ZFNs)	Protein	*FOK*I	20–40%	[41]
Transcription activator-like effector nuclease (TALEN)	Protein	*FOK*I	53.7% (double knockout) 78% TCR KO 78.8 & 81.2%	[34] [42]
CRISPR/Cas9	gRNA	Cas9	60%	[43]
			>80%	[38]
			~70%	[28]
			77% (CAR+ cells)	[30]
megaTAL Nucleases	Protein	Meganuclease	75%	[43]
Engineered I-CreI Homing endonuclease	Protein		>60%	[29]
